# Changes in Social Engagement for Incident Caregivers Compared to Controls

**DOI:** 10.1093/geroni/igaa057.2274

**Published:** 2020-12-16

**Authors:** Chelsea Liu, Marcela Blinka, Chanee Fabius, Virginia Howard

**Affiliations:** 1 Johns Hopkins University, Baltimore, Maryland, United States; 2 Johns Hopkins University, School of Medicine, Baltimore, Maryland, United States; 3 Johns Hopkins Bloomberg School of Public Health, Baltimore, Maryland, United States; 4 University of Alabama at Birmingham, Birmingham, Alabama, United States

## Abstract

Maintaining social engagement is important for the health and well-being of older adults who become caregivers. We assessed the association between incident caregiving and leisure satisfaction as well as the 10-year change in social network size among 245 incident caregivers and 248 matched controls. Multiple linear regression analyses were used to adjust for age, gender, race, education level, income, and geographic region. Compared to controls, incident caregivers had significantly lower levels of leisure satisfaction (p<0.01) and greater declines in total social network size (p<0.01). Incident caregivers and controls did not differ on the change in the number of social network members contacted monthly. Among incident caregivers, dementia caregivers and spouse caregivers had lower leisure satisfaction compared to non-dementia caregivers and non-spouse caregivers, respectively, but no differences were found on social network measures. Future studies should further examine social engagement among caregivers and its influence on their health outcomes.

